# Examination of a Viral Infection Mimetic Model in Human iPS Cell-Derived Insulin-Producing Cells and the Anti-Apoptotic Effect of GLP-1 Analogue

**DOI:** 10.1371/journal.pone.0144606

**Published:** 2015-12-11

**Authors:** Megu Yamaguchi Baden, Kenji Fukui, Yoshiya Hosokawa, Hiromi Iwahashi, Akihisa Imagawa, Iichiro Shimomura

**Affiliations:** Department of Metabolic Medicine, Graduate School of Medicine, Osaka University, Suita, Japan; University of Nantes, FRANCE

## Abstract

**Aims:**

Viral infection is associated with pancreatic beta cell destruction in fulminant type 1 diabetes mellitus. The aim of this study was to investigate the acceleration and protective mechanisms of beta cell destruction by establishing a model of viral infection in pancreatic beta cells.

**Methods:**

Polyinosinic:polycytidylic acid was transfected into MIN6 cells and insulin-producing cells differentiated from human induced pluripotent stem cells via small molecule applications. Gene expression was analyzed by real-time PCR, and apoptosis was evaluated by caspase-3 activity and TUNEL staining. The anti-apoptotic effect of Exendin-4 was also evaluated.

**Results:**

Polyinosinic:polycytidylic acid transfection led to elevated expression of the genes encoding IFNα, IFNβ, CXCL10, Fas, viral receptors, and IFN-inducible antiviral effectors in MIN6 cells. Exendin-4 treatment suppressed the elevated gene expression levels and reduced polyinosinic:polycytidylic acid-induced apoptosis both in MIN6 cells and in insulin-producing cells from human induced pluripotent stem cells. Glucagon-like peptide-1 receptor, protein kinase A, and phosphatidylinositol-3 kinase inhibitors counteracted the anti-apoptotic effect of Exendin-4.

**Conclusions:**

Polyinosinic:polycytidylic acid transfection can mimic viral infection, and Exendin-4 exerted an anti-apoptotic effect both in MIN6 and insulin-producing cells from human induced pluripotent stem cells.

## Introduction

Fulminant type 1 diabetes mellitus (FT1DM) is a severe subtype of type 1 diabetes characterized by extremely acute and severe insulin insufficiency as a result of almost complete destruction of the pancreatic beta cells even at clinical onset [1]. It is commonly observed in East Asia, where it accounts for approximately 20% of acute-onset type 1 diabetes cases in Japan [2] and 7.1% of all type 1 diabetes cases in South Korea [3]. It is likely that viral infection contributes to the pathogenesis of FT1DM. A nationwide survey in Japan revealed that 72% of FT1DM cases included a history of flu-like symptoms prior to onset [2]. Anti-enterovirus, anti-human herpesvirus 6, and anti-cytomegalovirus antibody levels are increased in some FT1DM patients [2]. In the pancreas of patients with FT1DM, enteroviral RNA was directly detected [4]. Recently, it was reported that viral infections may be a possible trigger in beta cell destruction even in type 1A diabetes, which was supposed to account for a major portion of type 1 diabetes cases [5]. Thus, an investigation of the mechanism of beta cell destruction via viral infection is important to clarify the pathophysiology of both FT1DM and type 1A diabetes.

Glucagon-like peptide-1 (GLP-1) is an incretin hormone with multiple physiological roles in pancreatic beta cells, including activation of insulin secretion, enhancement of insulin gene transcription and insulin biosynthesis, stimulation of beta cell proliferation, and inhibition of cytokine- [6–8] and lipotoxicity-induced [9] beta cell apoptosis. We hypothesized that exendin-4 (Ex4), GLP-1 analogue, could also inhibit beta cell apoptosis caused by viral infection. Initially we investigated the mechanism of beta cell destruction in a viral infectious situation and the protective effect of Ex4 by transfecting polyinosinic:polycytidylic acid (PIC) into MIN6 cells, a mouse-derived beta cell line [10]. PIC is a synthetic analogue of viral dsRNA [11], which is known to be a strong inducer of the innate immune responses against viral infection [12] and is often used to mimic viral infection both *in vivo* and *in vitro* [13–15]. Furthermore, we extended our study to include insulin-producing cells differentiated from human induced pluripotent stem (iPS) cells to establish a viral infection model of human pancreatic beta cells and to evaluate the anti-apoptotic effect of Ex4 on human insulin-producing cells.

## Materials and Methods

### Cell Culture

MIN6 cells, a mouse-derived beta cell line [10], were cultured at 37°C with 5% CO_2_ in DMEM (Sigma–Aldrich, St. Louis, MO, USA) containing 450 mg/dl glucose supplemented with 10% FBS (Sigma–Aldrich), 100 U/ml penicillin (Nacalai Tesque, Kyoto, Japan), 100 μg/ml streptomycin (Nacalai Tesque), and 100 μM 2-mercaptoethanol (Nacalai Tesque).

409B2 cells, a human iPS cell line derived from a healthy individual, were purchased from RIKEN Bioresource Centre Cell Bank (Ibaraki, Japan). 409B2 cells were cultured over the Mitomycin C-treated SNL feeder cells at 37°C with 5% CO_2_ in Primate ES medium (ReproCELL, Kanagawa, Japan) supplemented with 4 ng/ml recombinant human basic fibroblast growth factor (Wako, Osaka, Japan) and 500 U/ml penicillin/streptomycin (Life Technologies, Carlsbad, CA, USA). At 70–80% confluence, 409B2 cells were induced to insulin-producing cells using the differentiation protocol described previously [16]. Briefly, cells were first differentiated into endodermal cells expressing sex-determining region Y-box 17 (stage 1, 4 days), then into pancreatic progenitor cells expressing pancreatic and duodenal homeobox-1 (stage 2, 6 days), and finally into insulin-producing cells (stage 3, 12 days). The iPS cell study protocol was approved by the Ethics Committee of Osaka University.

### dsRNA transfection

The synthetic dsRNAs, PIC, were purchased from Sigma–Aldrich and used at a concentration of 10 μg/ml. Transfection of PIC was performed with Lipofectamine 2000 (Life Technologies). In the control study, only the Lipofectamine reagent was added to the medium. After MIN6 cells were cultured for 24 h in 12-well plates at a density of 5 × 10^5^ cells/well for the gene expression analysis and 1 × 10^6^ cells/well for the caspase-3 activity assay, cells were transfected with PIC according to the manufacturer’s instructions. Five hours later the medium was replaced with DMEM containing 10% FBS and cultured for 19 h before evaluation. For the immunocytochemistry analysis, MIN6 cells were cultured in an 8-well chamber slide (Thermo Scientific, Chicago, IL, USA) at a density of 2.5 × 10^5^ cells/well and transfected with PIC as described above. 409B2 cells were cultured in an eight-well chamber slide at a density of 1.5 × 10^5^ cells/well and induced to insulin-producing cells as described above. On day 12 at stage 3, the medium was replaced with Improved MEM Zinc Option medium (Life technologies) and cells were transfected with or without 10 μg/ml PIC. Five hours later, the medium was replaced with Improved MEM Zinc Option medium containing 1% growth factor reduced B27 and cultured for 19 h before evaluation.

### Gene Expression Analysis

RNA samples were isolated from MIN6 cells using the RNeasy® Micro Kit (Qiagen, Valencia, CA, USA). They were quantified and reverse transcribed using the High Capacity cDNA Reverse Transcription kit (Applied Biosystems, Foster City, CA, USA). Real-time PCR was performed on the 7900HT Fast Real Time PCR System (Applied Biosystems) using SYBR Green and compared with a standard curve after normalizing to the endogenous control (β-actin). The primer sequences for mouse IFNα, IFNβ, CXC chemokine ligand 10 (CXCL10), Fas, toll-like receptor 3 (TLR3), retinoic acid-inducible gene-I (RIG-I), melanoma differentiation-associated gene 5 (MDA5), laboratory of genetics and physiology 2 (LGP2), interferon stimulated gene 15 (ISG15), Mx GTPase pathway 1 (Mx1), 2′,5′-oligoadenylate-synthetase 1 (OAS1), protein kinase R (PKR), GLP-1 receptor (GLP-1R), and β-actin are listed in Table 1. The Taqman primer and probe for ISG15 was purchased from Applied Biosystems.

**Table 1:** PCR primers.

	Forward	Reverse
IFNα	TGTGTGATGCAGGAACCTCCT	GGTACACAGTGATCCTGTGG
IFNβ	CGTGGGAGATGTCCTCAACT	AGATCTCTGCTCGGACCACC
CXCL10	ATGACGGGCCAGTGAGAATG	TCAACACGTGGGCAGGATAG
Fas	AACCAGACTTCTACTGCGATTCTCC	TTGTATTGCTGGTTGCTGTGC
TLR3	GAAGAGCCACAGTGATAGATGG	AACTCTCCAGCAGAAGAGACAC
RIG-I	GGCTGAAAGCAAGGCTGATG	GACACTCTGGCTCTTCCTGA
MDA5	CAGAAGACAACACAGAATCAGACAC	GCCCATGTTGCTGTTATGTCC
LGP2	CCACGACCTGCTCATCTGTA	ACACTCGTCCACCACAATCA
Mx1	TGGACCCTGAAGGGGATAGG	GCTGTCTCCCTCTGATACGG
OAS1	ATGAGGGCCTCTAAAGGGGT	TAGGCTGGCAGCACATCAAA
PKR	GAAGTCACAGAGCCCCCAAA	TGCAGAGTTTTTAGCAGTCCT
GLP-1R	CACTTTCTTTCTCCGCCTTG	GGATGCAAACAGGTTCAGGT
β-actin	AGATTACTGCTCTGGCTCCTA	TCGTACTCCTGCTTGCTGAT

### Enzyme-linked immunosorbent assay (ELISA)

The protein levels of IFNα and IFNβ in MIN6 cells with and without PIC transfection were determined by ELISA (R&D Systems, Abingdon, UK). MIN6 cells were cultured in 6-well plates at a density of 2 × 10^6^ cells/well, and were lysed by freeze-thaw for three times, and measurements of the levels of IFNα and IFNβ were performed according to the manufacturer’s instructions.

### Ex4 and inhibitors treatment

Cells were pretreated with or without 10 nM or 100 nM Ex4 (Sigma–Aldrich) for 20 h, transfected with PIC transfection, and treated with or without Ex4 (10nM/100nM) again for 19 h before evaluation. In the experiments with inhibitors, cells were preincubated with 100 nM Exendin-(9–39) (Ex9, Sigma–Aldrich), 25 μM LY294002 (Cell Signaling, Danvers, MA, USA), or 5 μM H89 (Cell Signaling) for 30 min before treatment with 100 nM Ex4. The duration and concentration of Ex4 and inhibitor treatment were determined according to previous studies and used after ensuring that there was no cell toxicity.

### Immunocytochemistry

MIN6 cells and insulin-producing cells induced from 409B2 cells in an 8-well chamber slide were transfected with PIC as describe above and co-transfected with 80 nM FITC-coupled siRNA (SiGLO Red Transfection Indicator [TI], Thermo Scientific). After 24-h incubation, cells were washed with PBS and fixed with 4% PFA for 1 h at room temperature. After blocking with 5% donkey serum, TUNEL staining was performed using the *In Situ* Cell Death Detection Kit, POD (Roche Diagnostics, Tokyo, Japan) according to the manufacturer’s instructions, and nuclei were stained with Hoechst 33342 (1:1000, Life Technologies). For the analysis of 409B2 cells, insulin-positive cells were stained with guinea pig anti-insulin (primary antibody; 1:500, A0564, Dako, Glostrup, Denmark) and then Alexa Fluor 647 goat anti-guinea pig IgG (secondary antibody; 1:500, Life Technologies). IFNβ positive cells were stained with rabbit anti-IFNβ (primary antibody; 1:100, ab140211, Abcam, Cambridge, MA, USA) and Alexa Fluor 488 goat anti-rabbit IgG (secondary antibody; 1:1000, Life Technologies). The quantification of positive cells was performed using the fluorescence microscope BZ-X710 (Keyence, Osaka, Japan) and counting all cells in each of 12 fields (MIN6 cells) or 6 fields (iPS cells), which were randomly selected in a bright-field image.

### Caspase-3 activity assay

Caspase-3 activity was measured by APOPCYTO™ Caspase-3 Colorimetric Assay Kit (MBL, Nagoya, Japan) according to the manufacturer’s instructions. Briefly, cells were lysed using cell lysis buffer and incubated in 96-well plates with reaction buffer containing 10 mM DTT and caspase-3 substrate at 37°C for 18 h. The absorbances of the samples were measured at 400 nm using a SpectraMax 190 Microplate Reader (Molecular Devices, Sunnyvale, CA, USA), normalised to total protein, and presented as the relative intensity compared with the blank. Active caspase-3 content of MIN6 cells were also assessed using phycoerythrin (PE) Active Caspase-3 Apoptosis Kit by Becton, Dickinson and Company (BD) Biosciences Pharmingen (San Jose, CA, USA) according to the manufacturer’s instructions, and analysed by flow cytometry (BD FACS CantⅡFlow Cytometer, BD Biosciences).

### Statistical analysis

Data were analyzed using the Windows-based SPSS software (Version 22, IBM Co., Armonk, NY, USA) and expressed as mean ± SE. Differences between two groups were evaluated using an unpaired t-test, and more than three groups were evaluated using a one-way analysis of variance. All statistical significances were determined at p<0.05. All experiments were carried out at least three times, and reproducibility was confirmed.

## Results

### PIC induces cytokine, chemokine, viral receptor, and IFN-inducible antiviral effector gene expression and Ex4 suppresses PIC-induced cytokine and chemokine gene expression

**Fig 1:**
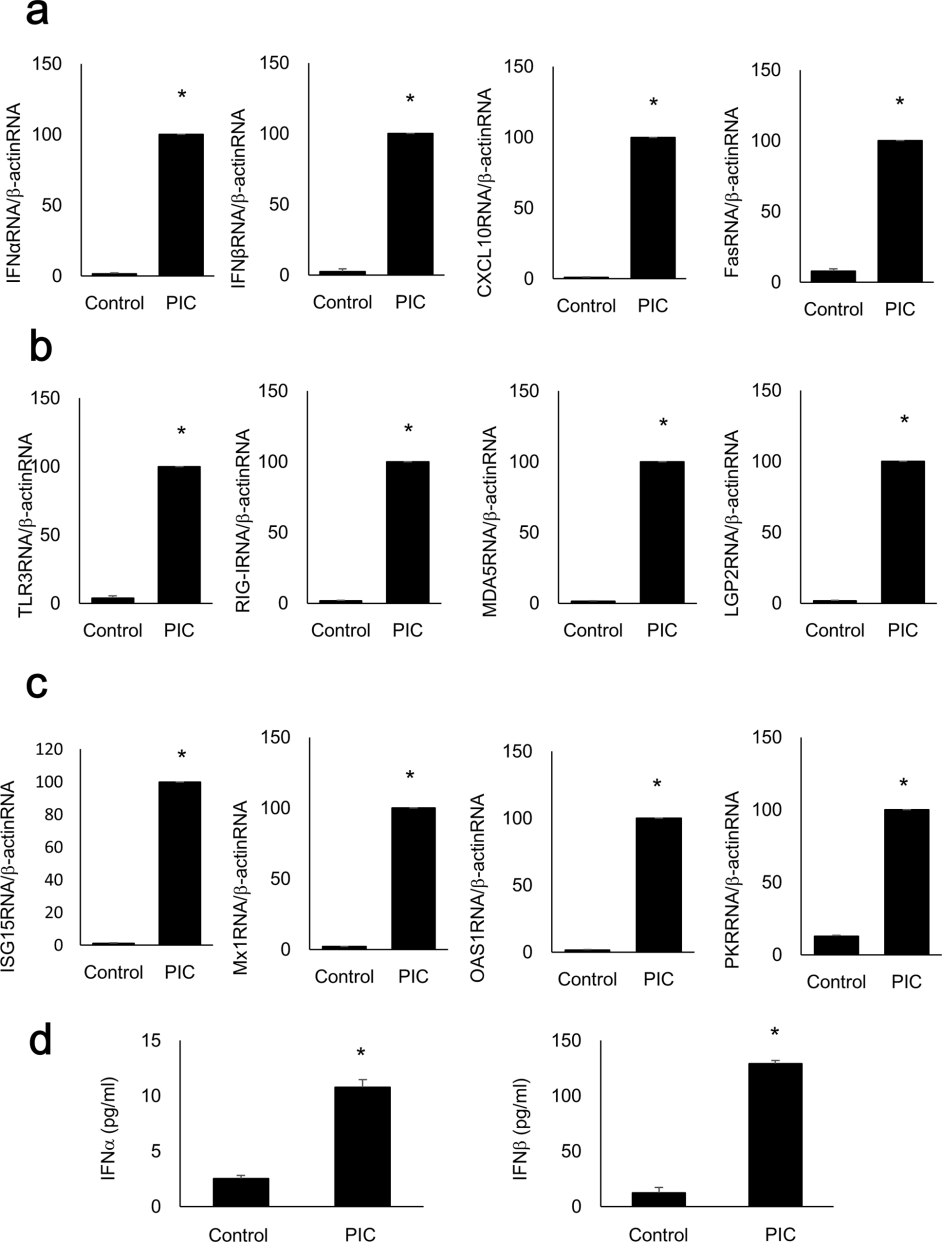
Expression levels of genes associated with viral infection increased following PIC transfection. Quantitative RT-PCR analysis was performed with RNA extracted from MIN6 cells with or without PIC transfection. IFNα, IFNβ, CXCL10, Fas (a), TLR3, RIG-I, MDA5, LGP2 (b), ISG15, Mx1, OAS1, and PKR (c) gene expressions were significantly increased compared with those of the control (Lipofectamine only) cells (n = 3). ELISA was also performed with cell lysates of MIN6 cells with or without PIC transfection. The levels of IFNα and IFNβ were significantly increased in PIC-transfected MIN6 cells compared with those in the control cells (Fig 1d, n = 3). The data were normalized to β-actin gene expression, with the relative gene expressions of the PIC-transfected cells arbitrarily set to 100. The error bars represent SE. The asterisk indicates significant difference (p<0.05).

**Fig 2:**
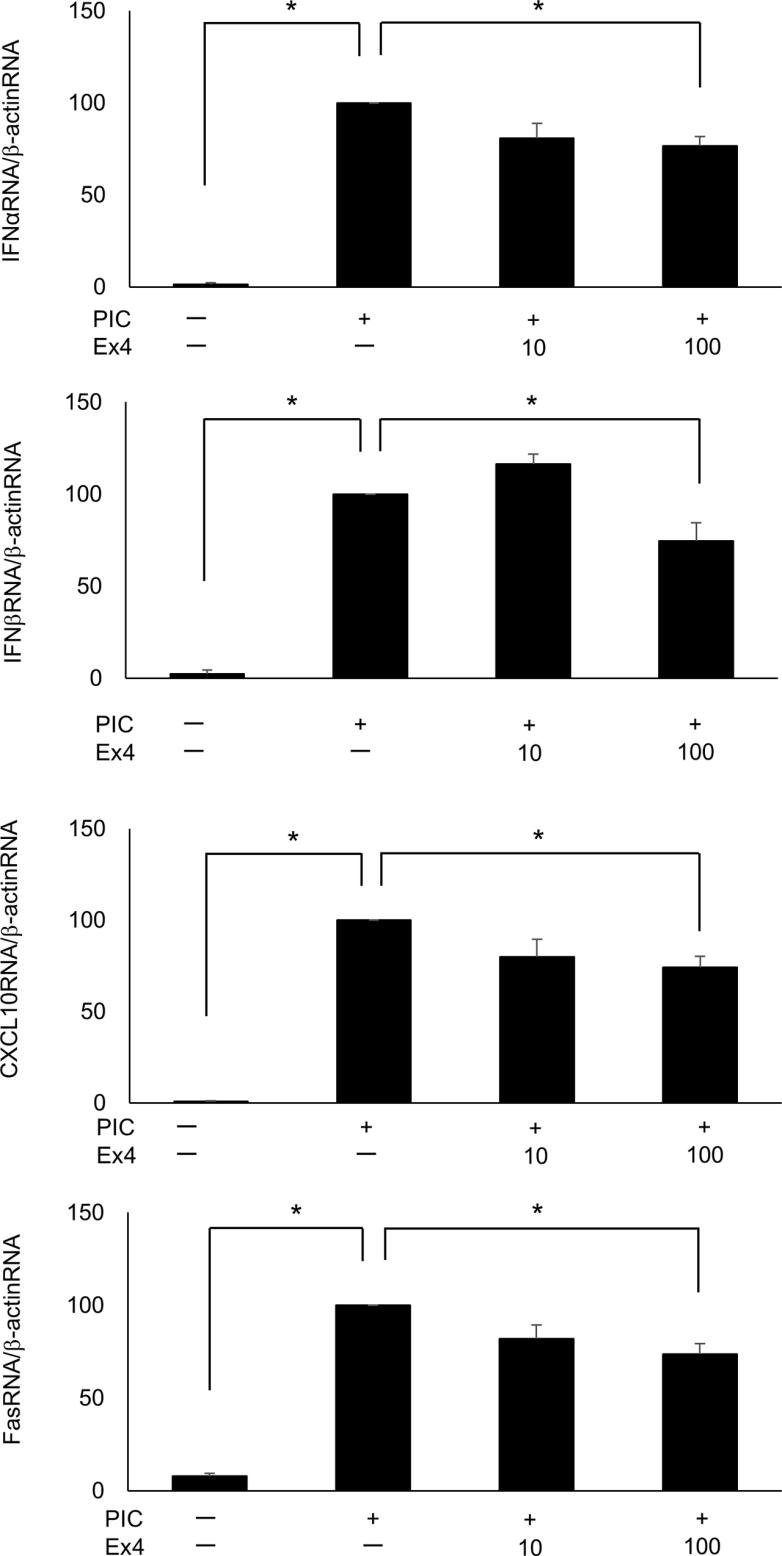
Ex4 inhibits the PIC-induced increase in cytokine and chemokine gene expression. Quantitative RT-PCR analysis of IFNα, IFNβ, CXCL10, and Fas was performed in PIC-transfected MIN6 cells with or without Ex4 (10 nM and 100 nM, n = 3). The data were normalized to β-actin gene expression, with the relative gene expressions of the PIC-transfected cells arbitrarily set to 100. The error bars represent SE. The asterisk indicates significant difference (p<0.05).

The expression levels of the genes encoding IFNα, IFNβ, CXCL10, and Fas were significantly increased following transfection of 10 μg/ml PIC compared with the control MIN6 cells (Fig 1A). Similarly, TLR3, RIG-I, MDA5, and LGP2 (Fig 1B), ISG15, Mx1, OAS1, and PKR gene expression levels (Fig 1C) were significantly increased in PIC-transfected MIN6 cells. The protein levels of IFNα and IFNβ measured by ELISA were also significantly increased by the transfection of 10 μg/ml PIC, compared with the control MIN6 cells (Fig 1D). Quantitative RT-PCR demonstrated that only adding 10 μg/ml PIC to the medium (without transfection) for 24 h did not alter IFNα, IFNβ, CXCL10, or Fas gene expression (S1 Fig, p = 0.052, 0.171, 0152, and 0.451, respectively). Exposure to 100 nM Ex4 significantly suppressed PIC-induced (p<0.05 vs. control cells) IFNα, IFNβ, CXCL10, and Fas gene expression (p<0.05 vs. PIC-transfected cells) in MIN6 cells (Fig 2).

### PIC transfection stimulates apoptosis while Ex4 significantly reduces PIC-induced apoptosis in MIN6 cells

**Fig 3:**
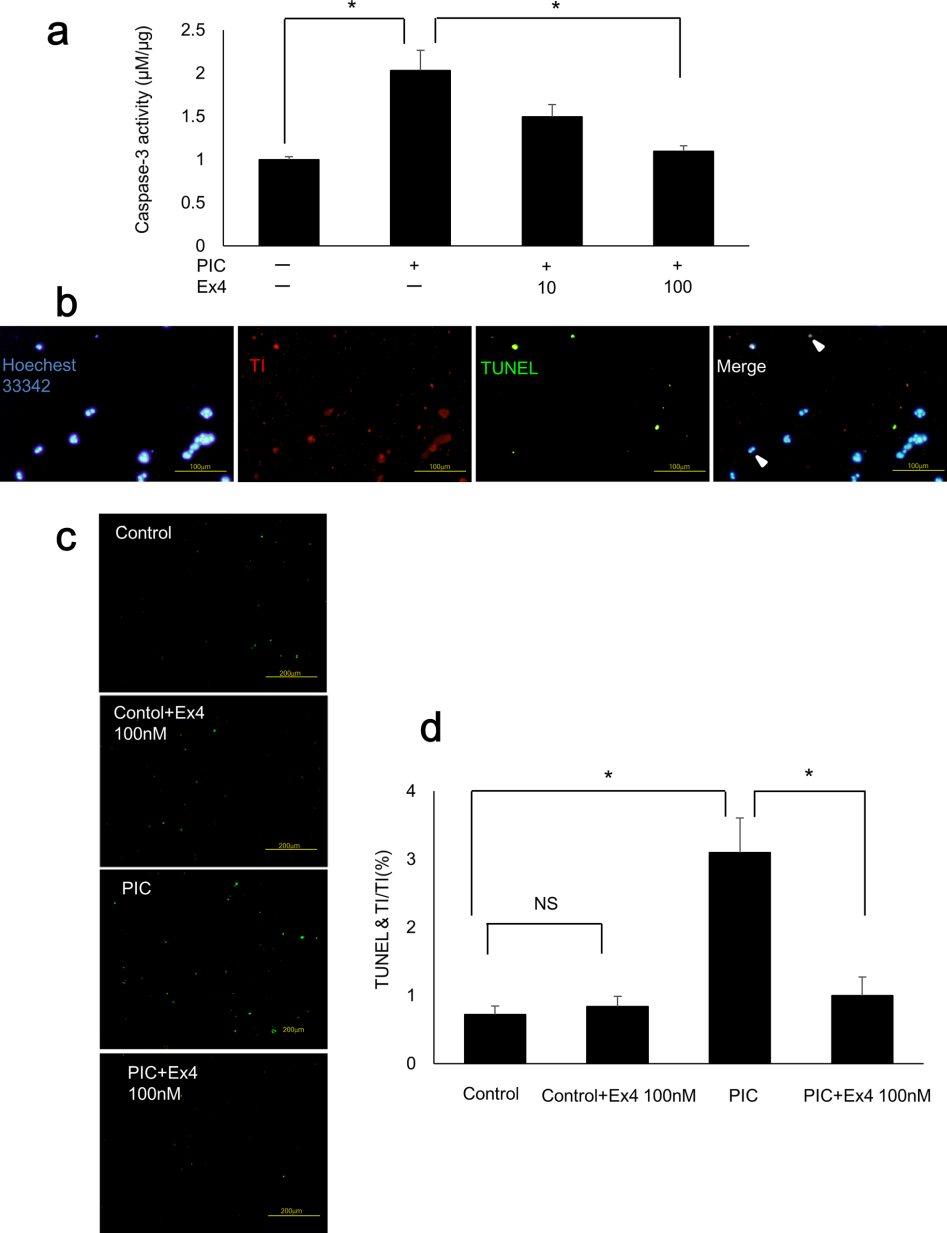
PIC transfection stimulated apoptosis while Ex4 treatment reduced PIC-induced apoptosis in MIN6 cells. (a) Caspase-3 activities of PIC-transfected cells with or without Ex4 (10 nM and 100 nM). The data were expressed as the caspase-3-to-protein content ratio, with that of the control cells arbitrarily set to 1. The error bars represent SE. The asterisk indicates significant difference (p<0.05). (b) TUNEL staining of MIN6 cells. Nuclei were detected in blue with Hoechst 33342, transfected cells in red with TI, and TUNEL-positive cells in green. Scale bars = 100 μm. (c) TUNEL staining of control MIN6 cells (Lipofectamine only) with or without 100nM Ex4 and PIC-transfected cells with or without 100 nM Ex4. Scale bars = 200 μm. (d) TUNEL-positive cells in TI-positive MIN6 cells. The error bars represent SE. The asterisk indicates significant difference (p<0.05). NS represents no significant difference.

Caspase-3 activity was significantly increased in PIC-transfected MIN6 cells (p<0.05 vs. control cells) and significantly decreased following exposure to 100 nM Ex4 (p<0.05 vs. PIC-transfected cells; Fig 3A). Next, we detected nuclei in blue using Hoechst 33342, transfected cells in red using TI, and TUNEL-positive cells in green (Fig 3B). The TUNEL-positive rate was calculated in transfected cells. The transfection efficacy was 84.6% in control cells, 31.5% in PIC-transfected cells, and 30.1% in PIC-transfected cells treated with Ex4 (100 nM). Transfection efficacy did not significantly differ between cells treated with PIC alone and cells treated with both PIC and 100 nM Ex4 (S2 Fig, p = 0.907). The TUNEL-positive rate was significantly higher in PIC-transfected MIN6 cells (p<0.05 vs. control cells) than in control cells. Furthermore, this effect was significantly decreased following exposure to 100 nM Ex4 (p<0.05 vs. PIC-transfected cells; Fig 3C and 3D). Ex4 treatment of control cells did not affect the TUNEL-positive rate compared with that in untreated control cells (p = 0.282, Fig 3C and 3D).

### Ex4 treatment reduces PIC-induced apoptosis via the GLP-1 receptor and both the protein kinase A (PKA) and phosphatidylinositol 3-kinase (PI-3K) pathways

**Fig 4:**
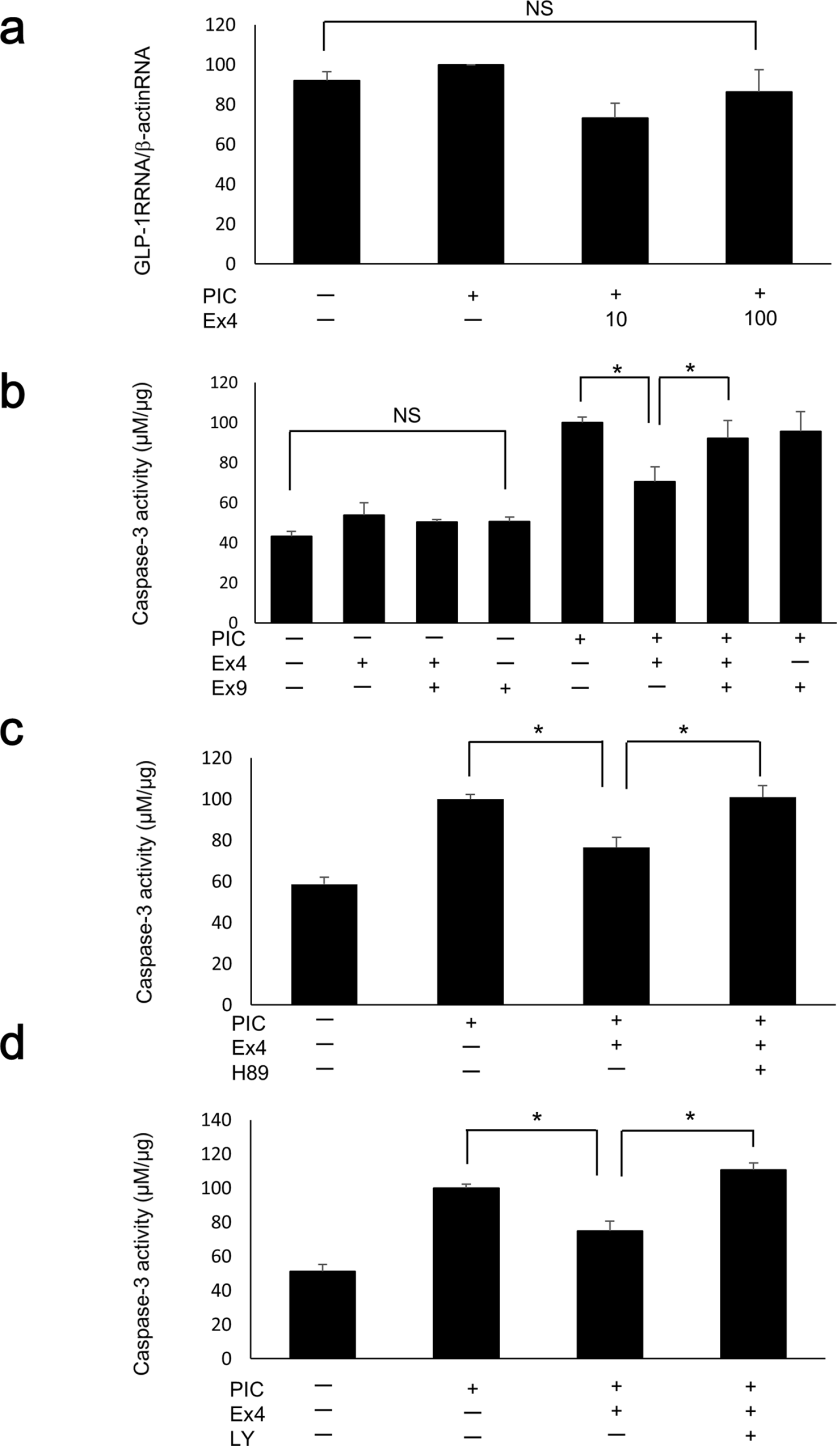
Ex4 mitigated PIC-induced apoptosis via the GLP-1 receptor and both the PKA and PI-3K pathways. (a) Quantitative RT-PCR analysis of the GLP-1 receptor was performed in PIC-transfected MIN6 cells with or without Ex4 (10 nM and 100 nM, n = 3). The data were normalized to β-actin gene expression, with the relative gene expressions of the PIC-transfected cells arbitrarily set to 100. The error bars represent SE. (b–d) Caspase-3 activities of PIC-transfected MIN6 cells treated with 100 nM Ex4 and Ex9 (b), H89 (c), or LY294002 (d). The data are expressed as the caspase-3-to-protein content ratio, with that of the PIC-transfected cells without Ex4/Ex9, H89, or LY294002 arbitrarily set to 100. The error bars represent SE. The asterisk indicates significant difference (p<0.05). NS represents no significant difference.

GLP-1 receptor gene expression did not differ among control cells, PIC-transfected cells, PIC-transfected cells with 10 nM Ex4, and PIC-transfected cells with 100 nM Ex4 (p = 0.129; Fig 4A). The PIC-induced reduction in caspase-3 activity following exposure to 100 nM Ex4 was significantly inhibited by pre-treatment with the GLP-1 receptor antagonist Ex9 (100 nM; Fig 4B), demonstrating that the anti-apoptotic effect of Ex4 is mediated via the GLP-1 receptor. Ex4 and Ex9 had no significant effect on caspase-3 activity under control (Lipofectamine only) conditions (p = 0.206; Fig 4B). Similarly, the decrease in PIC-induced caspase-3 activity following exposure to 100 nM Ex4 was significantly inhibited following treatment with both the PKA inhibitor H89 (Fig 4C) and the PI-3K inhibitor LY294002 (Fig 4D). We also confirmed the similar changes of active caspase-3 at protein level by flow cytometry (S3 Fig). The population of cells that were positive for active caspase-3 was increased by PIC transfection, and reduced by the exposure to 100nM Ex4. And the reduction was inhibited by the treatment with Ex9, H89, and LY294002. H89 and LY294002 had no significant effect on caspase-3 activity under control conditions (S4 Fig, p = 0.185 and p = 0.067, respectively).

### PIC transfection increased IFNβ in insulin-producing cells differentiated from human iPS cells

**Fig 5:**
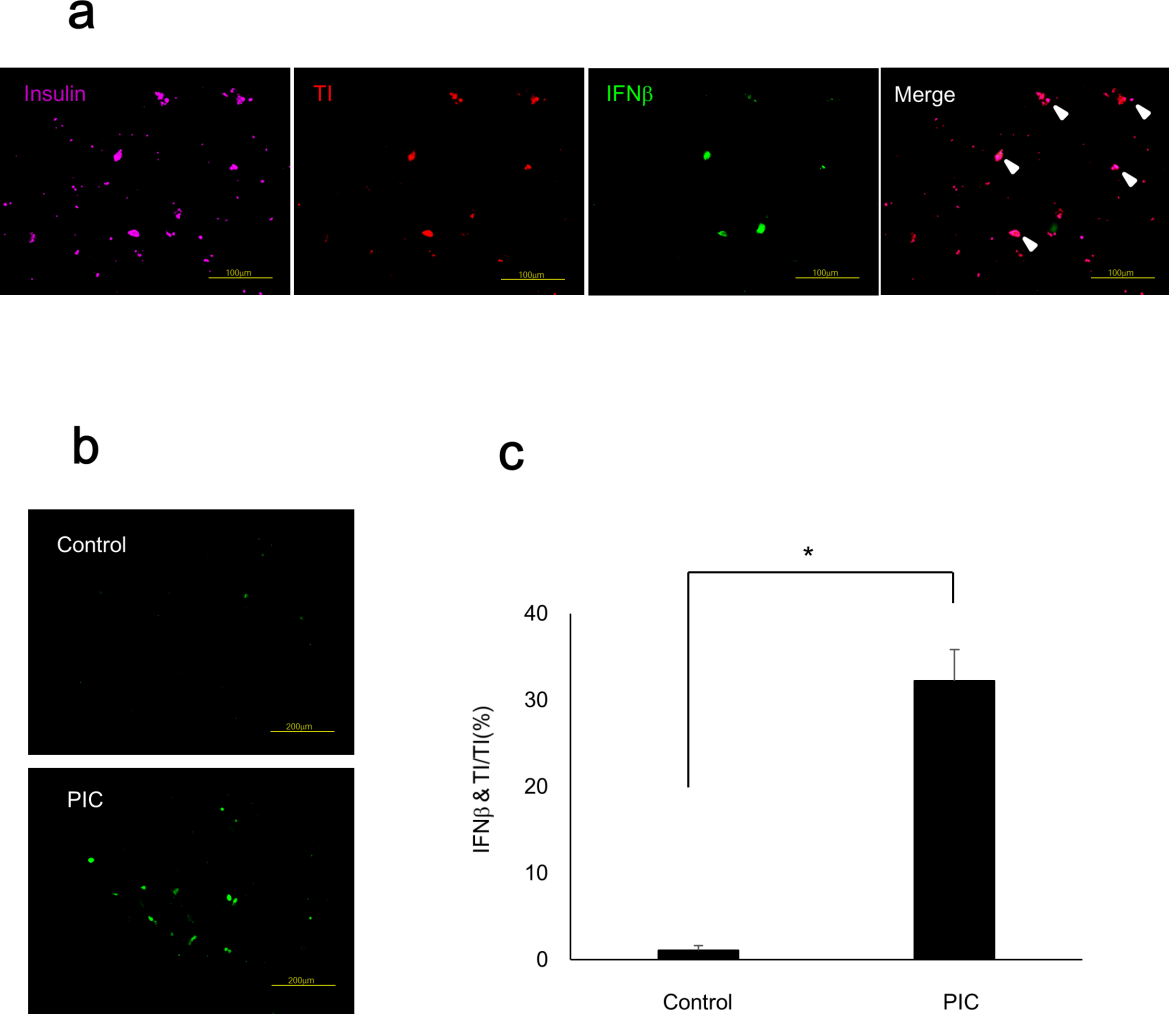
PIC transfection increased IFNβ in insulin-producing cells differentiated from human iPS cells. (a) Immunostaining of insulin-producing cells differentiated from human iPS cells. Insulin-positive cells were detected in purple, transfected cells in red with TI, and IFNβ-positive cells in green. Scale bars = 100 μm. (b) IFNβ staining of control cells and PIC-transfected cells. Scale bars = 200 μm. (c) IFNβ-positive ratio in TI-positive insulin-producing cells. The error bars represent SE. The asterisk indicates significant difference (p<0.05).

The proportion of insulin-producing cells differentiated from 409B2 cells, human iPS cells from a healthy individual, was 8.1% ± 0.3%, which was comparable with a previous report [16]. We detected insulin-producing cells in purple, transfected cells in red (TI), and IFNβ-positive cells in green to evaluate the IFNβ-positive ratio of PIC-transfected insulin-producing cells (Fig 5A). The IFNβ-positive ratio was significantly increased in PIC-transfected insulin-producing cells (p<0.05 vs. control cells) as shown in Fig 5B and 5C.

### PIC transfection induces apoptosis while Ex4 treatment significantly reduces PIC-induced apoptosis in insulin-producing cells differentiated from human iPS cells

**Fig 6:**
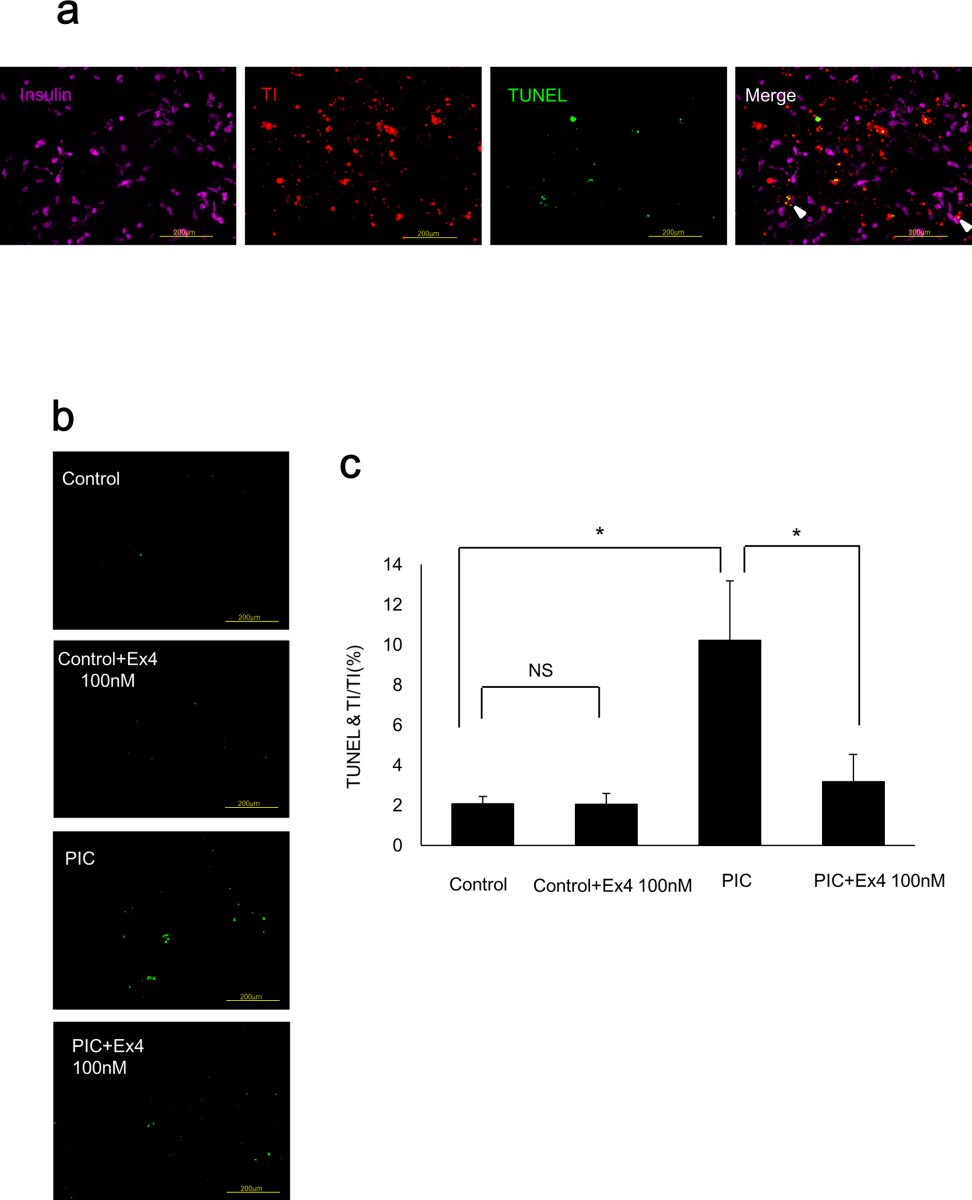
PIC stimulated apoptosis while Ex4 reduced PIC-induced apoptosis in insulin-producing cells from human iPS cells. (a) Immunostaining of insulin-producing cells differentiated from human iPS cells. Insulin-positive cells were detected in purple, transfected cells in red with TI, and TUNEL-positive cells in green. Scale bars = 200 μm. (b) TUNEL staining of control cells with or without 100nM Ex4 and PIC-transfected cells with or without 100 nM Ex4. (c) TUNEL-positive cells in TI-positive insulin-producing cells. The error bars represent SE. The asterisk indicates significant difference (p<0.05). NS represents no significant difference.

We detected insulin-producing cells in purple, transfected cells in red (TI), and TUNEL-positive cells in green to evaluate the apoptosis ratio of PIC-transfected insulin-producing cells (Fig 6A). The TUNEL-positive rate was significantly increased in PIC-transfected insulin-producing cells (p<0.05 vs. control cells) and significantly decreased following exposure to 100 nM Ex4 (p<0.05 vs. PIC-transfected cells; Fig 6B and 6C). Ex4 treatment of control cells did not alter the TUNEL-positive rate compared with that in untreated control cells (p = 0.483, Fig 6B and 6C).

## Discussion

The aim of the present study was to establish a human model of beta cell destruction and to identify a novel treatment in a situation of viral infection. We performed PIC transfections in mouse pancreatic beta cells and insulin-producing cells differentiated from human iPS cells of a healthy individual. PIC transfection increased the expression of genes that are stimulated by viral infection and the protein levels of IFNα and IFNβ, and induced apoptosis. Furthermore, Ex4 treatment protected against PIC-induced apoptosis. These results suggest that PIC transfection can be used to establish a human viral infection model, and that Ex4 can be a beneficial protective agent against type 1 diabetes mellitus development.

We confirmed that PIC transfection increased the expression of the genes encoding cytokines, chemokines, viral receptors, and IFN-inducible antiviral effectors, which have been previously reported to be stimulated by viral infection. When viral infection occurs, TLR3 in endosomes and RIG-I, MDA5, and LGP2 in the cytoplasm recognize the dsRNA. TLR3 recruits the adaptor TIR-domain-containing adapter-inducing interferon-β(TRIF) and activates NF-κB and IRF resulting in the induction of type I IFNs (IFNα and IFNβ). RIG-I and MDA5 are RNA helicases containing caspase activation and recruitment domains (CARD), which recruits CARD adaptor inducing IFN-β (Cardif) and activates NF-κB and IRF [17]. LGP2 is a helicase related to RIG-I and MDA5 but lacking CARDs and functioning as a negative regulator of host defence [18]. CXCL10, which reacts with the chemokine (C-X-C motif) receptor 3 on Th1 cells and stimulates T cell and NK cell chemotaxis [19], and Fas were also expressed following viral infection [20, 21]. Moreover, after type I IFN binds to IFN receptors, four effector pathways are activated to initiate an antiviral response: the ISG 15 pathway, the Mx pathway, the OAS pathway, and the PKR pathway. These pathways individually block viral transcription, degrade viral RNA, inhibit translation, and modify protein function to control viral replication [22]. In the present study, PIC induces these same pathways, including reactions that have been reported to occur in FT1DM [4]. Therefore, PIC transfection is a useful method for mimicking viral infection involved in FT1DM development.

We demonstrated that apoptosis of beta cells in a viral infectious situation might occur as a result of the beta cell antiviral reaction. Caspase-3 activity and the TUNEL-positive rate in PIC-transfected MIN6 cells and insulin-producing cells were significantly increased compared with those of control cells. The progression of beta cell destruction in FT1DM is extremely rapid [1]. It is possible that apoptosis of beta cells directly by viral infection may also play a role in the progression of FT1DM. In contrast, previous studies on FT1DM reported that macrophages and T cells infiltrated the pancreatic beta cells, suggesting their involvement in FT1DM beta cell destruction [4, 23].

Ex4 may play a protective role in the progression of beta cell destruction in a viral infectious situation. Ex4 inhibited PIC-induced beta cell apoptosis both in MIN6 cells and in human insulin-producing cells. Previous studies have reported that GLP-1 and its analogue inhibit apoptosis by acting against the pro-inflammatory cytokines, such as IL-1β, IFNγ, and TNF-α, which are secreted by immune cells, in isolated rat beta cells [24, 25], MIN6 cells, and isolated human islets [26]. In this study, we focused on IFNα, IFNβ, CXCL10, and Fas, which were produced by beta cells in a viral infectious situation, and demonstrated that Ex4 suppressed cytokine gene expression and inhibited beta cell apoptosis. We further demonstrated that PKA and PI-3K inhibitors counteracted the anti-apoptotic effect of Ex4, indicating that both PKA and PI-3K play a role in inhibiting beta cell apoptosis. PKA and PI-3K are reported to be primary effectors in the GLP-1 receptor-dependent intracellular signal transduction pathways in the pancreatic beta cell that inhibit apoptosis [6]. It has been reported that the activation of cAMP/PKA leads to the activation of Bcl-2 and Bcl-XL [6], induction of the Akt-PKB growth and survival pathway [27, 28], and dephosphorylation of eukaryotic translation initiation factor 2α [29]. Activation of PI-3K results in the activation of Akt/PKB and inhibits the activities of nuclear factor-κB [30], Foxo1 [31], and caspase [6]. As PIC induces apoptosis through the activation of nuclear factor-κB and the phosphorylation of eukaryotic translation initiation factor 2α [32], it is reasonable that both PKA and PI-3K exert a protective effect against PIC-induced apoptosis.

This is the first study in which we transfected PIC into insulin-producing cells differentiated from human iPS cells to establish a human viral infection model. The IFNβ-positive rate was significantly increased in PIC-transfected insulin-producing cells. The TUNEL-positive rate was also significantly increased in PIC-transfected insulin-producing cells, and significantly decreased following exposure to Ex4. These data confirmed that it was possible to imitate viral infection by transfecting PIC into insulin-producing cells from human iPS cells as well as MIN6 cells. Previous studies have used islets that were isolated from donors as an alternative way to evaluate the mechanism of human beta cell destruction following viral infection [33, 34]. Because the number of donor pancreases is limited, human iPS cells are advantageous in that they can be provided easily and indefinitely. Another advantage is that insulin-producing cells induced from patients’ own iPS cells are specific to their disease. Thus, the application of this method to iPS cells of FT1DM or type 1A patients will clarify their own characteristic features concerning disease pathophysiology. One limitation of the pancreatic beta cell differentiation protocols, including the protocol we used in this study, is that they are not fully established yet. Thus, the obtained differentiated cells are reported to be immature beta cells, which resemble fetal beta cells [35]. However, differentiation protocols are improving, and some groups have recently reported a method for producing more differentiated beta cells that possess glucose-stimulated insulin secretory capacity [36, 37]. In the near future, it might be possible to apply the protocol of this study to completely mature beta cells differentiated from iPS cells.

In conclusion, we demonstrated that PIC transfection can mimic viral infection both in MIN6 cells and insulin-producing cells differentiated from human iPS cells. Furthermore, Ex4 treatment exerted an anti-apoptotic effect against PIC transfection. The application of this study to disease-specific iPS cells, such as from type 1 diabetes patients, will clarify the pathophysiological mechanism of disease onset and progression and may contribute to the development of novel therapeutics.

## Supporting Information

S1 Fig Quantitative RT-PCR demonstrated that only adding 10 μg/ml PIC to the medium for 24 h did not alter IFNα, IFNβ, CXCL10, or Fas gene expression. The data were normalized to β-actin gene expression, with the relative gene expressions of the control cells arbitrarily set to 1. The error bars represent SE. NS represents no significant difference.
(TIF)

S2 Fig Transfection efficacy did not differ between PIC-transfected cells and PIC-transfected cells with Ex4. **The transfection efficacy of control cells was significantly higher than that of PIC-transfected cells.** The bar graphs display TI-positive cells in Hoechst 33342-positive MIN6 cells. The error bars represent SE. The asterisk indicates significant difference (p<0.05). NS represents no significant difference.
(TIF)

S3 Fig Flow cytometric analysis of apoptotic and non-apoptotic populations for active caspase-3. **The population of cells that were positive for active caspase-3 was increased by PIC transfection, and reduced by the exposure to 100nM Ex4. And the reduction was inhibited by the treatment with Ex9, H89, and LY294002.** MIN6 cells were permeabilized, fixed, stained for active caspase-3 and analysed by flow cytometry according to the manufacturer’s instructions. The numbers in upper right corners showed the percentage of cells that were positive for active caspase-3 staining.
(TIF)

S4 Fig H89 and LY294002 had no significant effect on caspase-3 activity under control conditions. The data are expressed as the caspase-3-to-protein content ratio, with that of the PIC-transfected cells without Ex4, H89, or LY294002 arbitrarily set to 100. The error bars represent SE. NS represents no significant difference.
(TIF)
