# Supplementary figures and images for: Examination of a Viral Infection Mimetic Model in Human iPS Cell-Derived Insulin-Producing Cells and the Anti-Apoptotic Effect of GLP-1 Analogue
Source: PLoS One. 2015 Dec 11;10(12):e0144606. doi: 10.1371/journal.pone.0144606 (PMC4676675; doi:10.1371/journal.pone.0144606)

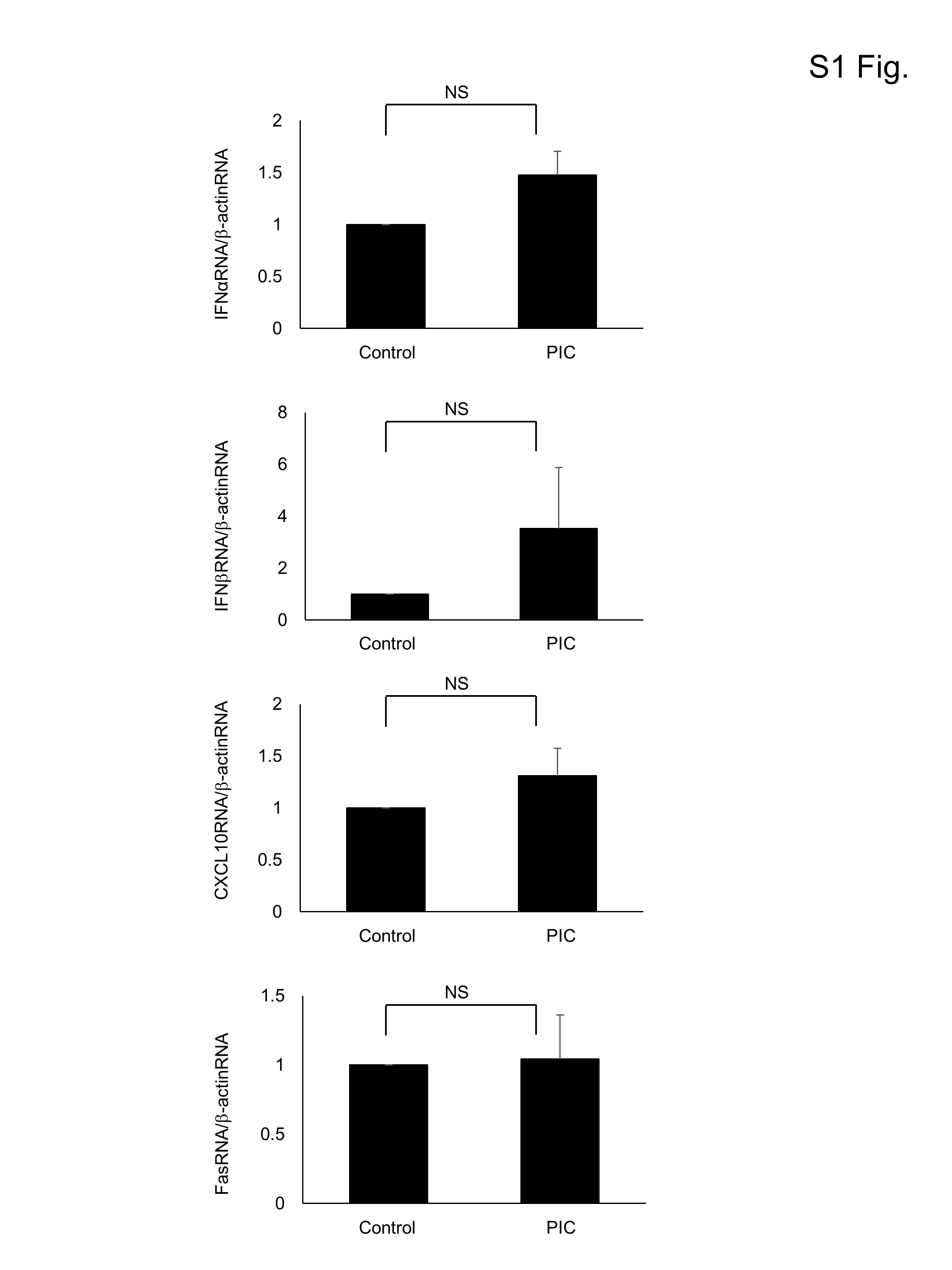

Supplement: S1 Fig — The data were normalized to β-actin gene expression, with the relative gene expressions of the control cells arbitrarily set to 1. The error bars represent SE. NS represents no significant difference. (TIF) [file pone.0144606.s001.tif]

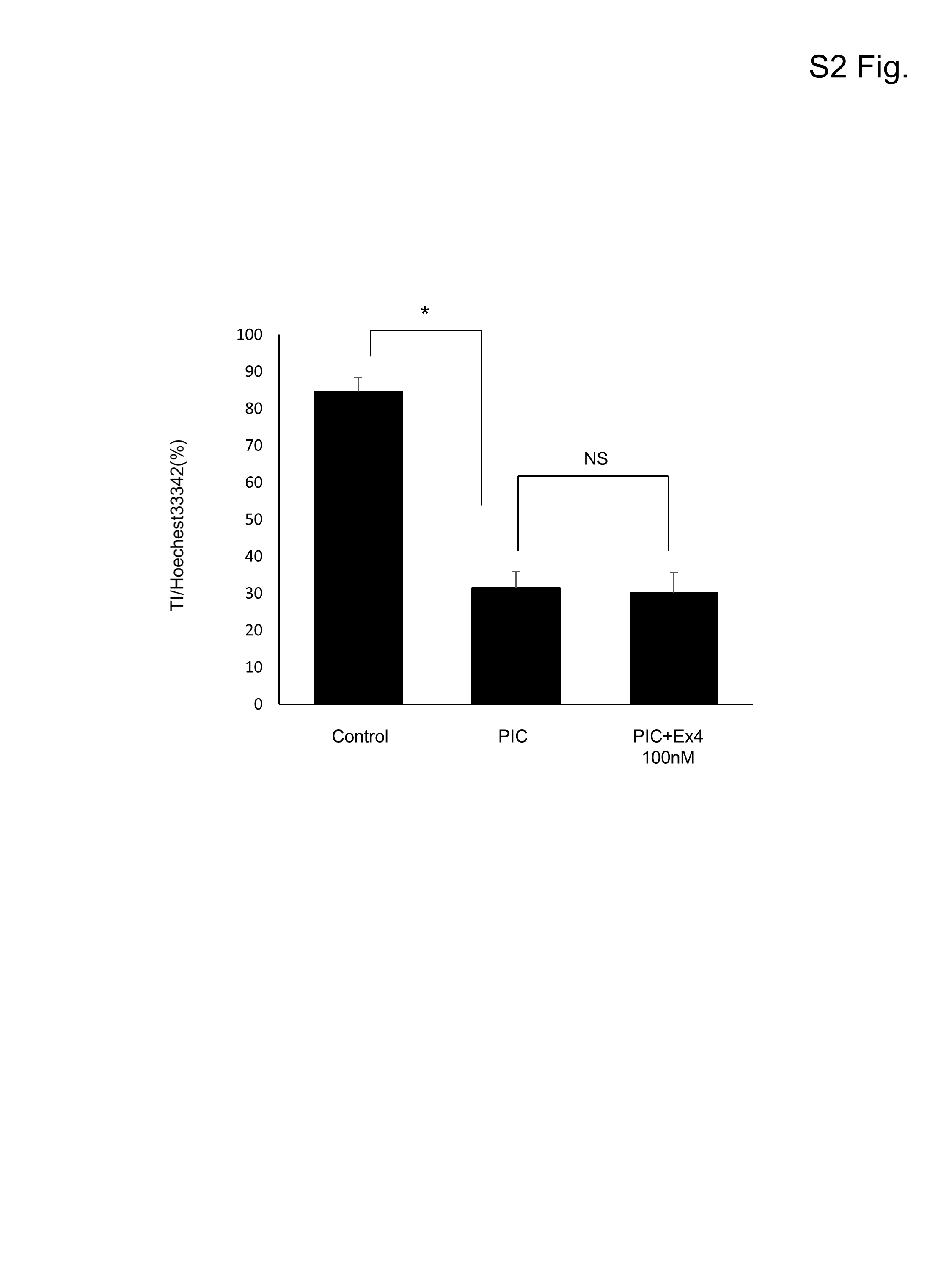

Supplement: S2 Fig — The transfection efficacy of control cells was significantly higher than that of PIC-transfected cells. The bar graphs display TI-positive cells in Hoechst 33342-positive MIN6 cells. The error bars represent SE. The asterisk indicates significant difference (p<0.05). NS represents no significant difference. (TIF) [file pone.0144606.s002.tif]

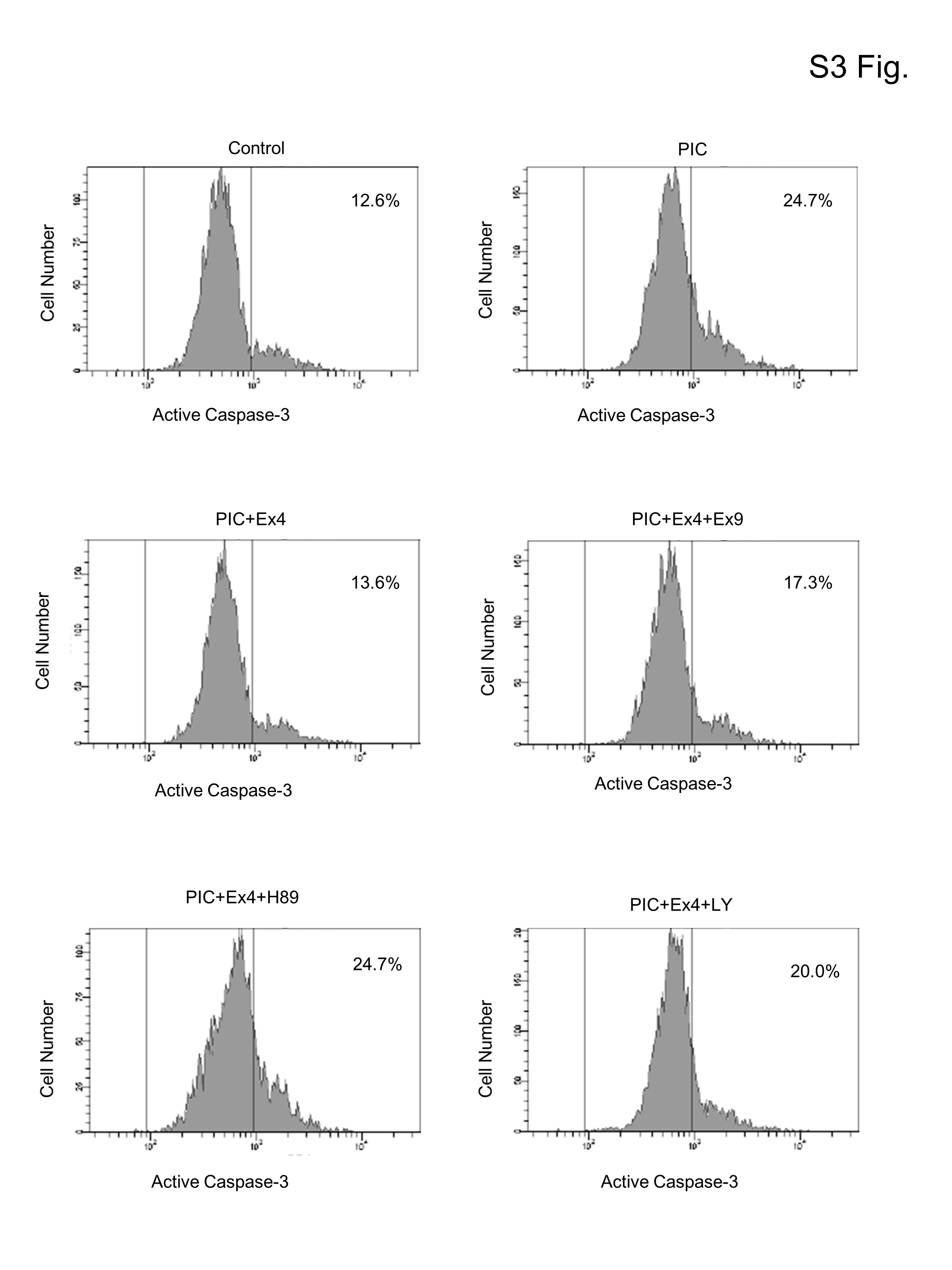

Supplement: S3 Fig — The population of cells that were positive for active caspase-3 was increased by PIC transfection, and reduced by the exposure to 100nM Ex4. And the reduction was inhibited by the treatment with Ex9, H89, and LY294002. MIN6 cells were permeabilized, fixed, stained for active caspase-3 and analysed by flow cytometry according to the manufacturer’s instructions. The numbers in upper right corners showed the percentage of cells that were positive for active caspase-3 staining. (TIF) [file pone.0144606.s003.tif]

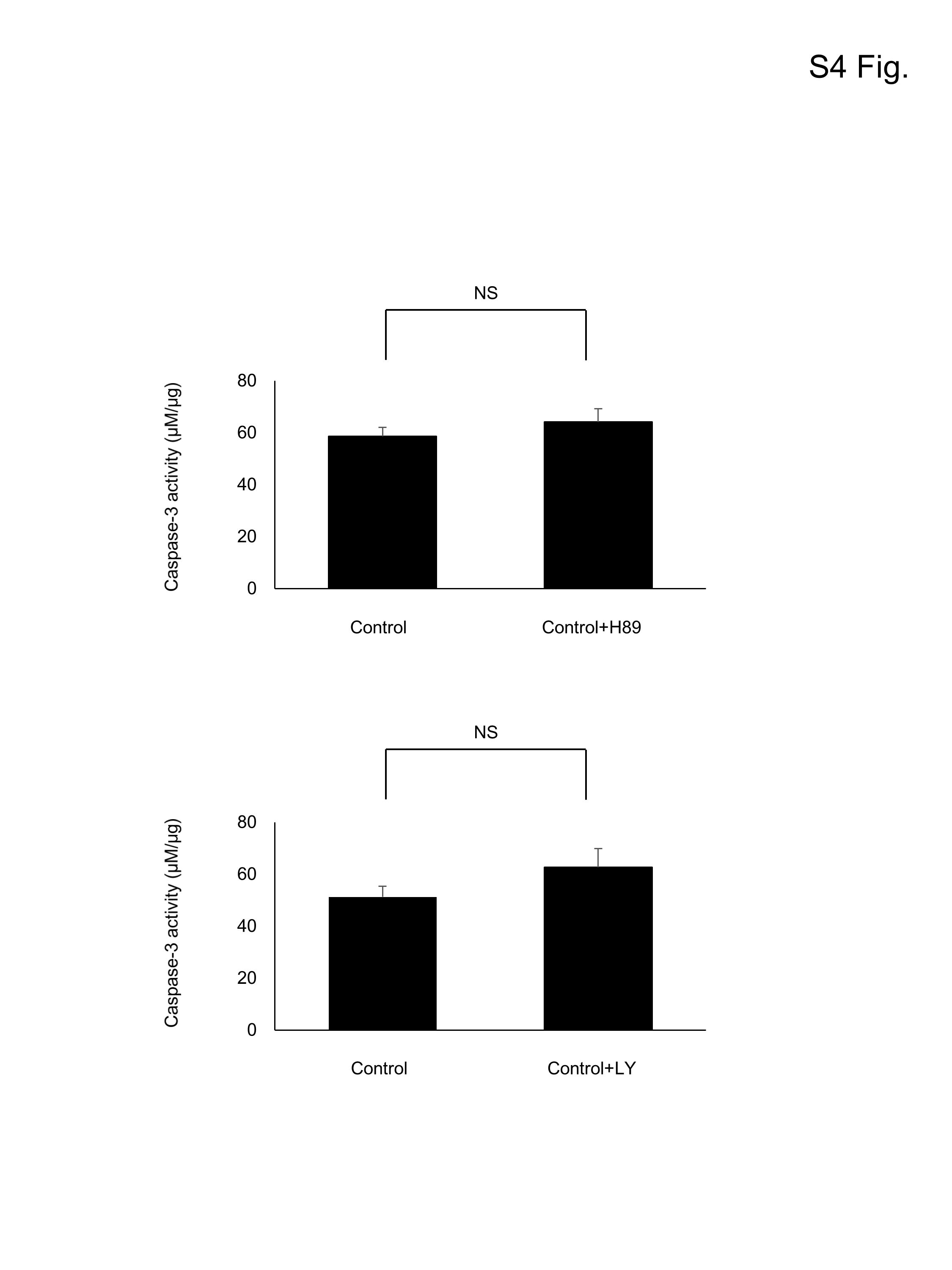

Supplement: S4 Fig — The data are expressed as the caspase-3-to-protein content ratio, with that of the PIC-transfected cells without Ex4, H89, or LY294002 arbitrarily set to 100. The error bars represent SE. NS represents no significant difference. (TIF) [file pone.0144606.s004.tif]
